# The Observer

**Published:** 1896-07

**Authors:** 


### BOOK NOTICES.

THE OBSERVER: An illustrated monthly magazine for students and lovers of nature. Published monthly by E. F. Bigelow, Portland, Conn. Subscription, $1.00 per year. Ten cents a single copy.

The July number of this instructive and elegant journal is before us. Among other articles we find one on "The Anatomy of the May Beetle," illustrated, with diagrams of its several parts; one on "The Study of the Grasshopper;" an entertaining essay on the character and habits of a song-bird called the "Prothonotary Warbler;" another interesting article is "The Pine Grosbeak in Northern New York." These are followed by a series of articles upon astronomy. One of these collects from that "Thesaurus of human wisdom, the Bible," all the references to the stars of heaven; another is upon "Kepler, the Legislator of the Heavens;" another is upon "Danger from Comets." Most interesting, especially to the physician, is the series of lessons in Practical Bacteriology in the microscopical department of the journal. From these articles it will be seen that the journal interests every order, from the physician to the wandering school-boy roaming through field and wood.

